# Supplementary material for: Reconciling Mining with the Conservation of Cave Biodiversity: A Quantitative Baseline to Help Establish Conservation Priorities
Source: PLoS One. 2016 Dec 20;11(12):e0168348. doi: 10.1371/journal.pone.0168348 (PMC5173368; doi:10.1371/journal.pone.0168348)
Supplement: S1 Dataset — (ZIP) [file pone.0168348.s002.zip › Taxa/Serra Norte/SN_2013/N5S/Lista N5S-44.pdf]

## CAVIDADE N5S-0044/0046

| Classe | Ordem | Fam/Outros | Gên/Outros | Espécie | S-U | U-S | Zon. |
|---|---|---|---|---|---|---|---|
| Annelida | Hirudinea | | | sp.1 | | 4 | E |
| Annelida | Oligochaeta | | | jovem | 4 | 6 | E/P |
| Arachnida | Acari | (Oribatida) | | sp.8 | X | | E |
| Arachnida | Acari | Cunaxidae | | sp.1 | X | | P |
| Arachnida | Acari | Laelapidae | | sp.69 | X | | P |
| Arachnida | Acari | Rhagidiidae | | sp.3 | | X | P |
| Arachnida | Acari | Veigaiidae | | sp.1 | X | X | E/P |
| Arachnida | Araneae | Araneidae | | jovem | X | X | E |
| Arachnida | Araneae | Ochyroceratidae | | jovem | X | X | E/P |
| Arachnida | Araneae | Oonopidae | *Neoxyphinus* | sp.2 | X | X | E |
| Arachnida | Araneae | Pholcidae | *Mesabolivar* | *aurantiacus* | | X | P |
| Arachnida | Araneae | Pholcidae | *Mesabolivar* | sp.1 | X | X | E |
| Arachnida | Araneae | Pisauridae | | jovem | 1 | 2 | E/P |
| Arachnida | Araneae | Salticidae | | jovem | X | | P |
| Arachnida | Araneae | Scytodidae | *Scytodes* | sp.1 | | 2 | E |
| Arachnida | Araneae | Theridiosomatidae | *Plato* | sp.1 | X | X | E |
| Arachnida | Opiliones | Cosmetidae | *Roquettea* | *singularis* | 1 | | |
| Arachnida | Opiliones | Sclerosomatidae | *Geaya* | sp.1 | 1 | | E |
| Arachnida | Opiliones | Sclerosomatidae | *Prionostema* | sp.1 | 4 | 12 | E |
| Arachnida | Ricinulei | Ricinoididae | *Cryptocellus* | jovem | | 1 | P |
| Chilopoda | Scolopendromorpha | Cryptopidae | *Cryptops* | jovem | 1 | 2 | E/P |
| Chilopoda | Scolopendromorpha | Scolopocryptopidae | *Newportia* | sp.1 | 2 | | E |
| Diplopoda | Polydesmida | Chelodesmidae | | jovem | | 1 | P |
| Diplopoda | Polydesmida | Fuhrmannodesmidae | | sp.1 | | X | P |
| Diplopoda | Polydesmida | Pyrgodesmidae | | jovem | X | | E |
| Entognatha | Collembola | Entomobryidae | | sp.1 | X | | E |
| Entognatha | Collembola | Entomobryidae | | sp.6 | | X | E/P |
| Entognatha | Collembola | Entomobryidae | | sp.7 | | X | E |
| Entognatha | Collembola | Isotomidae | | sp.1 | | X | P |
| Entognatha | Collembola | Paronellidae | | sp.1 | X | X | E/P |
| Entognatha | Collembola | Paronellidae | | sp.4 | | X | P |
| Entognatha | Collembola | Poduromorpha | | sp.1 | X | | P |
| Entognatha | Diplura | Campodeidae | | sp.1 | X | X | E/P |
| Entognatha | Diplura | Japygidae | | sp.1 | | X | E |
| Insecta | Blattodea | | | jovem | 1 | 3 | E/P |
| Insecta | Coleoptera | Carabidae | | sp.3 | X | X | E/P |
| Insecta | Coleoptera | Dytiscidae | | sp.5 | X | X | E/P |
| Insecta | Coleoptera | Hydrophilidae | | sp.7 | X | | E |
| Insecta | Coleoptera | Staphylinidae | Pselaphinae | sp.2 | | X | P |
| Insecta | Coleoptera | Staphylinidae | Pselaphinae | sp.17 | X | | P |
| Insecta | Coleoptera | Staphylinidae | | sp.13 | | X | E |
| Insecta | Coleoptera | Staphylinidae | | sp.16 | X | | P |
| Insecta | Coleoptera | Staphylinidae | | sp.27 | X | | E |
| Insecta | Diptera | Cecidomyiidae | Cecidomyiinae | sp. | | X | E |
| Insecta | Diptera | Ceratopogonidae | | sp. | | X | P |
| Insecta | Diptera | Culicidae | *Culex* | sp. | X | | E/P |
| Insecta | Diptera | Drosophilidae | *Drosophila* | *eleonorae* | | X | |
| Insecta | Diptera | Phoridae | Metopininae | sp. | X | | E |
| Insecta | Diptera | Phoridae | Phorinae | sp. | X | | P |

| | | | | | | | |
|---|---|---|---|---|---|---|---|
| Insecta | Diptera | Psychodidae | *Breviscapus* | sp. | X | | E |
| Insecta | Diptera | Psychodidae | Phlebotominae | sp. | X | | E |
| Insecta | Diptera | Sciaridae | | sp. | | X | E |
| Insecta | Diptera | Sphaeroceridae | | sp. | X | | E |
| Insecta | Diptera | Tipulidae | Tipulinae | sp. | X | X | E |
| Insecta | Ephemeroptera | Leptophlebiidae | | sp.1 | X | | E/P |
| Insecta | Heteroptera | Enicocephalidae | | sp. | X | | P |
| Insecta | Heteroptera | Notonectidae | *Buenoa* | sp. | X | X | E/P |
| Insecta | Heteroptera | Tingidae | | sp. | | X | P |
| Insecta | Heteroptera | Veliidae | *Microvelia* | jovem | X | | E |
| Insecta | Heteroptera | Veliidae | *Paravelia* | sp. | X | X | E/P |
| Insecta | Hymenoptera | Diapriidae | | sp.1 | X | X | E/P |
| Insecta | Hymenoptera | Formicidae | *Crematogaster* | sp.1 | X | X | E/P |
| Insecta | Hymenoptera | Formicidae | *Eurhopalothrix* | sp.1 | X | X | P |
| Insecta | Hymenoptera | Formicidae | *Gnamptogenys* | sp.1 | X | | E |
| Insecta | Hymenoptera | Formicidae | Myrmicinae | sp.1 | | X | E |
| Insecta | Hymenoptera | Formicidae | *Odontomachus* | sp.1 | X | | E |
| Insecta | Hymenoptera | Formicidae | *Pachycondyla* | sp.1 | | X | P |
| Insecta | Hymenoptera | Formicidae | *Pachycondyla* | sp.2 | X | X | P |
| Insecta | Hymenoptera | Formicidae | *Paratrechina* | sp.1 | | X | E |
| Insecta | Hymenoptera | Formicidae | *Paratrechina* | sp.2 | | X | E |
| Insecta | Hymenoptera | Formicidae | *Pheidole* | sp.2 | X | X | E/P |
| Insecta | Hymenoptera | Formicidae | *Pheidole* | sp.3 | | X | E |
| Insecta | Hymenoptera | Formicidae | *Pheidole* | sp.4 | X | | E/P |
| Insecta | Hymenoptera | Formicidae | *Solenopsis* | sp.1 | X | | P |
| Insecta | Hymenoptera | Formicidae | *Trachymyrmex* | sp.1 | X | X | E/P |
| Insecta | Isoptera | Rhinotermitidae | *Heterotermes* | sp. | X | | E |
| Insecta | Isoptera | Termitidae | *Nasutitermes* | sp. | X | X | E/P |
| Insecta | Lepidoptera | | | jovem | X | | P |
| Insecta | Orthoptera | Acrididae | | sp. | 1 | | E |
| Insecta | Orthoptera | Gryllidae | Nemobiinae | jovem | | 1 | E |
| Insecta | Orthoptera | Phalangopsidae | *Paraclodes* | jovem | 5 | 4 | E |
| Insecta | Orthoptera | Phalangopsidae | *Phalangopsis* | sp. | 2 | 11 | |
| Insecta | Psocoptera | Pseudocaeciliidae | *Phallocaecilius* | sp.1 | X | | E |
| Gastropoda | Pulmonata | Subulinidae | | sp.2 | | X | P |
| Gastropoda | Pulmonata | Subulinidae | | sp.5 | X | | P |
| Gastropoda | Pulmonata | Systrophiidae | *Happia* | sp. | X | X | E/P |
| Malacostraca | Isopoda | Philosciidae | | sp.1 | X | X | E/P |
| Malacostraca | Isopoda | Philosciidae | | sp.2 | X | | P |
| Nematoda | | | | sp.1 | X | | E |
| Amphibia | Anura | | | sp. | 7 | x | E/P |
| Reptilia | Squamata | Sauria | | sp. | | 1 | E |
